# Overexpression of the Wheat (*Triticum aestivum* L.) *TaPEPKR2* Gene Enhances Heat and Dehydration Tolerance in Both Wheat and *Arabidopsis*

**DOI:** 10.3389/fpls.2018.01710

**Published:** 2018-11-23

**Authors:** Xinshan Zang, Xiaoli Geng, Kexiang He, Fei Wang, Xuejun Tian, Mingming Xin, Yingyin Yao, Zhaorong Hu, Zhongfu Ni, Qixin Sun, Huiru Peng

**Affiliations:** State Key Laboratory for Agrobiotechnology, Key Laboratory of Crop Heterosis and Utilization (MOE), Beijing Key Laboratory of Crop Genetic Improvement, China Agricultural University, Beijing, China

**Keywords:** *TaPEPKR2*, heat stress, dehydration stress, PEP carboxylase kinase-related kinase, wheat, *Arabidopsis*

## Abstract

Wheat (*Triticum aestivum* L.) yield and quality are adversely affected by heat, drought, or the combination of these two stresses in many regions of the world. A phosphoenolpyruvate carboxylase kinase-related kinase gene, *TaPEPKR2*, was identified from our previous heat stress-responsive transcriptome analysis of heat susceptible and tolerant wheat cultivars. Based on the wheat cultivar Chinese Spring genome sequence, *TaPEPKR2* was mapped to chromosome 5B. Expression analysis revealed that *TaPEPKR2* was induced by heat and polyethylene glycol treatment. To analyze the function of *TaPEPKR2* in wheat, we transformed it into the wheat cultivar Liaochun10, and observed that the transgenic lines exhibited enhanced heat and dehydration stress tolerance. To examine whether *TaPEPKR2* exhibits the same function in dicotyledonous plants, we transformed it into *Arabidopsis*, and found that its overexpression functionally enhanced tolerance to heat and dehydration stresses. Our results imply that *TaPEPKR2* plays an important role in both heat and dehydration stress tolerance, and could be utilized as a candidate gene in transgenic breeding.

## Introduction

Heat and drought stress and their combination during the growing season are major environmental factors affecting the production and quality of wheat worldwide. A 5.5% decrease in global wheat yields was caused by heat stress between 1980–2008 (Lobell et al., 2011), while drought stress caused considerable yield loss and high economic costs in more than 50% of wheat cultivation areas (Ashraf, 2010). Therefore, research into the genetic mechanism of heat and drought stress tolerance is getting increasingly important.

Plants have developed a range of response mechanisms to adjust to abiotic stress, especially molecular responses to maintain normal activities (Shinozaki and Dennis, 2003; Ashraf, 2010; Mittler et al., 2012; Qu et al., 2013). Genes responding to abiotic stress are essential for enhancing abiotic stress tolerance, and an understanding of these is crucial to developing abiotic stress-tolerant crops.

Protein kinases regulate key aspects of cellular function, including responses to external signals. In *Arabidopsis*, around 4% of predicted genes encode typical protein kinases (Hrabak et al., 2003). Phosphoenolpyruvate carboxylase kinase (PPCK)-related kinases (PEPKRs) are unique to plants and belong to the CDPK-SnRK superfamily (Hrabak et al., 2003). PPCKs are calcium-independent protein kinases that function in crassulacean acid metabolism and C4 plants (Vidal and Chollet, 1997; Nimmo et al., 2001; Agetsuma et al., 2004). Predicted PEPKR proteins in *Arabidopsis* contain both N- and C-terminal extensions with no similarity to the non-catalytic domains of other kinases in this superfamily (Hrabak et al., 2003). Thus far, their functions remain unknown.

In this study, we isolated the coding and promoter region of *TaPEPKR2*, and analyzed the expression pattern of *TaPEPKR2* under heat and dehydration stress conditions. Finally, a transgenic approach was used to investigate the function of *TaPEPKR2* in both wheat and *Arabidopsis* under heat and dehydration stress conditions.

## Materials and Methods

### Plant Materials, Growth Conditions, and Stress Treatments

The common wheat variety “TAM107,” which has a heat-tolerant phenotype and was released by Texas A&M University in 1984, was used for gene cloning and expression analysis. Growth conditions and stress treatments of wheat were as previously described (Zang et al., 2017a,b). Briefly, the sprouted seeds were grown on moistened filter paper at 22°C/18°C (day/night), 12 h/12 h (light/dark), and 60% humidity in a growth chamber. For high-temperature treatments, seedlings were transferred to another growth chamber maintained at 40°C. For dehydration stress treatments, water was replaced by PEG-6000 (20%). Leaves were collected from the seedlings at different time points, frozen immediately in liquid nitrogen and stored at -80°C for RNA isolation.

Common wheat cultivar Liaochun10 (LC10) and *Arabidopsis thaliana* ecotype Col-0 was used for genetic transformation.

### Cloning and Sequence Analysis

Total RNA was extracted from 7-day-old seedlings using TRIzol reagent (Invitrogen), and purified RNA was treated with DNase I. Subsequently, 2 μg of total RNA was reverse transcribed by M-MLV reverse transcriptase according to the instruction (Promega). Based on the probe sequence (Ta.10701.1.S1_at), a pair of gene-specific primers *TaPEPKR2*-L/R was used to amplify *TaPEPKR2*. Primer sequences are listed in Supplementary Table S1 (1, 2).

Database searches of nucleotide and deduced amino acid sequences of the *TaPEPKR2* homologs were analyzed by NCBI/GenBank/Blast. Sequence alignment and similarity comparisons were performed by DNAMAN software. The functional domains of *TaPEPKR2* were identified by SMART programs^1^.

### Expression Pattern Analysis of *TaPEPKR2* in Wheat

Quantitative real-time PCR (RT-qPCR) was performed to determine the relative expression level of *TaPEPKR2* with specific primers. The 2^-ΔΔC^_T_ method (Livak and Schmittgen, 2001) was used to quantify the relative expression of *TaPEPKR2*, and the wheat *β-actin* gene was used as a reference. Each experiment was repeated three times independently. Supplementary Table S1 (3, 4, 7, 8) lists the RT-qPCR primers.

### Transgenic Constructs of *TaPEPKR2* and Genetic Transformation in Wheat

The coding DNA sequence (CDS) of *TaPEPKR2* (Supplementary File S1) driven by the maize ubiquitin promoter was inserted into the binary vector pBract806. The resulting expression constructs were utilized for genetic transformation. LC10 immature embryos were utilized for wheat transformation via the particle bombardment method. The presence of a *TaPEPKR2* transgene was verified by PCR using primers listed in Supplementary Table S1 (5, 6). In total, five transgenic events (T1–T5) were produced and three lines (T3–T5) with higher expression levels were selected for further analysis.

### Thermotolerance Assay in Wheat

The thermotolerance assay in wheat was performed as previously described (Zang et al., 2017a). Seeds of LC10 and *TaPEPKR2* transgenic lines were grown in pots containing potting soil under the above-mentioned conditions. 5-day-old seedlings were transferred to a growth chamber at 45°C for 18 h, typically beginning at 09:00 h, and were then shifted to 22°C for recovery. The phenotypes were photographed 5 days after the treatment.

### Generation of *TaPEPKR2* Transgenic *Arabidopsis* Plants

The CDS of *TaPEPKR2* was amplified and cloned into the binary vector pB2GW7 using the gateway method. *Agrobacterium tumefaciens* strain GV3101 containing this binary construct was used to transform *Arabidopsis* plants by the floral dip method. Transformants were selected on 1/2 MS medium containing Basta (125 μL/L), followed by PCR amplification of positive clones. In total, seven transgenic lines (L1–L7) were produced and three lines (L1, L3, and L5) were selected for further analysis.

### Thermotolerance Assay in *Arabidopsis*

The thermotolerance assay in *Arabidopsis* was performed as previously described (Zang et al., 2017a,b). Surface-sterilized seeds of WT and *TaPEPKR2* transgenic lines were sown on MS solid medium. The plated 7-day-old seedlings were exposed to 45°C for 120 min in an illuminated growth chamber, then shifted to 22°C to the previous day/night cycle for 5–7 days recovery. Phenotypes before and after heat treatment were photographically documented.

### Ion Leakage Assay

Electrolyte leakage was measured as previously described (Camejo et al., 2005; Zang et al., 2017a,b). Leaf segments of uniform maturity were cut into disks and washed three times with de-ionized water to eliminate external residues. Six disks were placed in test tube flasks with 20 mL of de-ionized water and incubated at 42°C for 1 h. After incubation at room temperature for 24 h, the conductivity of the solutions was determined with a Horiba Twin Cond B-173 conductivity meter (HORIBA Ltd., Kyoto, Japan) and noted as T1. Next, the samples were boiled for 15 min to kill the tissues, followed by incubation at room temperature for 24 h. The conductivities of the solutions were then recorded as T2. Ion permeability was calculated as T1/T2. The experiment was repeated three times independently. Student’s *t*-test in Microsoft excel was used to determine the presence of significant differences (^∗^*P* < 0.05, ^∗∗^*P* < 0.01).

### Dehydration Tolerance Assay

In *Arabidopsis*, 10-day-old seedlings of WT and *TaPEPKR2* transgenic lines germinated on MS medium solidified with 0.8% agar were planted in identical pots, which contained 30 g mixed soil (vermiculite: humus = 1:1) and were added the same volume of water. Then, they were cultured in the greenhouse under optimum growth conditions (16-h-light/8-h-dark cycle, 150 μmol m^-2^ s^-1^, 22°C/18°C, 60% humidity). For dehydration tolerance assay, seedlings were subjected to water deprivation for 25 days, and then re-watered for about ∼1 week. In wheat, seeds of LC10 and *TaPEPKR2* transgenic lines were planted in water. 5-day-old seedlings of WT and *TaPEPKR2* transgenic lines were placed in water and 20% PEG conditions for 5 days. Student’s *t*-test in Microsoft excel was used to determine the presence of significant differences (^∗^*P* < 0.05).

## Results

### Cloning of a *TaPEPKR2* Gene From TAM107

**FIGURE 1:**
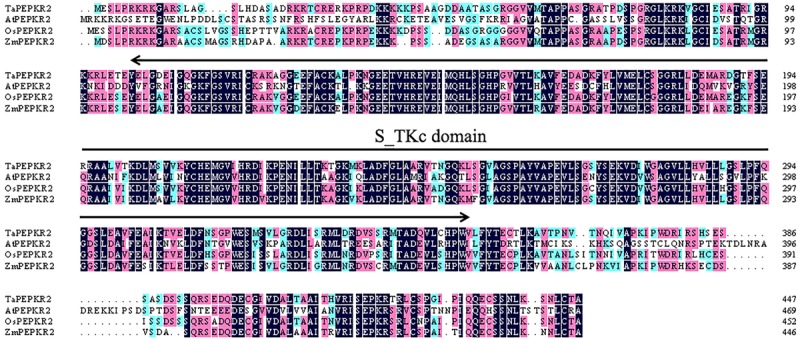
Sequence alignment of *TaPEPKR2* and orthologous genes from *Arabidopsis*, rice, and maize. Regions corresponding to the S_TKc domain are indicated by the SMART program.

Microarray analysis with the Affymetrix GeneChip^®^ Wheat Genome Array previously indicated that probe “Ta.10701.1.S1_at” was induced 2^2.73^-fold after 40°C treatment for 1 h (Qin et al., 2008). Based on this probe sequence, we cloned the open reading frame (ORF) of *TaPEPKR2* from wheat cultivar “TAM107” (previously named *TaSTK*, GenBank Accession No. GU213488.1). The complete ORF of *TaPEPKR2* is 1347 bp and encodes a polypeptide of 448 amino acid residues. Based on the wheat cultivar Chinese Spring genome sequence, *TaPEPKR2* was mapped to chromosome 5B. The protein sequence showed homology with PEPKR2 family members from other plant species, including *Zea mays* (ZmPEPKR2, 81.46%), *Oryza sativa* (OsPEPKR2, 81.98%), and *A. thaliana* (AtPEPKR2, 53.18%) (Figure 1). SMART analysis indicated that the amino acid sequence of TaPEPKR2 possessed a S_TKc motif, with both N- and C-terminal extensions (Figure 1 and Supplementary Figure S1).

### *TaPEPKR2* Is Induced by Heat and Dehydration Stress Treatment

**FIGURE 2:**
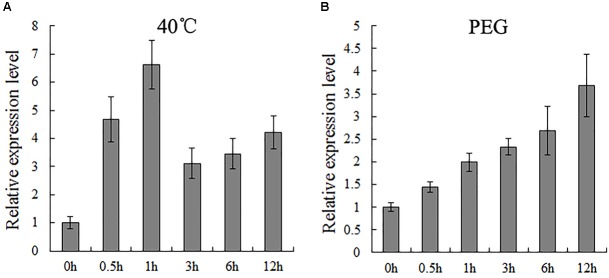
Relative expression of *TaPEPKR2* under heat **(A)** and dehydration **(B)** stress conditions, as determined by RT-qPCR. Data represent the mean of three replicates ± SD.

Relative expression levels of *TaPEPKR2* in wheat under heat and 20% PEG stress conditions were determined by RT-qPCR using gene-specific primers (Figure 2). The relative expression of *TaPEPKR2* increased and peaked at 1 h after heat treatment at 40°C and then decreased, but increased mRNA abundance was maintained (Figure 2A). Following treatment with 20% PEG, *TaPEPKR2* expression increased gradually and peaked at 12 h (Figure 2B). These results indicate that *TaPEPKR2* can be induced by heat and 20% PEG treatment.

### Overexpression of *TaPEPKR2* in Wheat Conferred Heat Tolerance at the Seedling Stage

To understand the function of *TaPEPKR2*, we transformed it into wheat cultivar LC10 under the control of the maize ubiquitin promoter by particle bombardment. A total of five transgenic lines were analyzed over T_1_ and T_2_ generations by PCR analysis with specific corresponding primers. Three lines (L3, L4, and L5) that exhibited *TaPEPKR2* up-regulation in shoots at the seedling stage (Supplementary Figure S2) were selected for further analysis.

**FIGURE 3:**
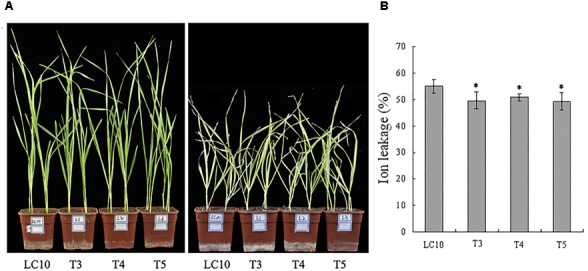
Heat tolerance assay of *TaPEPKR2* transgenic wheat seedlings. **(A)** Phenotypes of 10-day-old LC10 and three *TaPEPKR2* transgenic lines before heat treatment (left). 5-day-old LC10 and three *TaPEPKR2* transgenic wheat lines were treated at 45°C for 18 h, then recovered at 22°C for 5 days. Images were taken post-recovery (right). **(B)** Ion leakage assay of the transgenic seedlings in panel **(A)** after heat treatment. The symbol “^∗^” indicates significance at *P* < 0.05.

To examine the applicability of *TaPEPKR2* for thermotolerance transgenic breeding, we characterized the phenotypes of *TaPEPKR2* transgenic wheat at various developmental stages. Under optimum growth conditions, no visible difference was found between transgenic and WT plants (Figure 3A). However, after heat treatment and the recovery stage, WT plants wilted slightly more rapidly than *TaPEPKR2* transgenic lines (Figure 3A). Electrolyte leakage is an indicator to reflect heat stress-induced membrane injury. Thus, we evaluated electrolyte leakage with detached leaves after heat treatment. The results showed that transgenic lines exhibited significantly reduced electrolyte leakage compared with LC10 (Figure 3B).

### *TaPEPKR2* Overexpression in Wheat Enhanced Tolerance to Dehydration Stress

**FIGURE 4:**
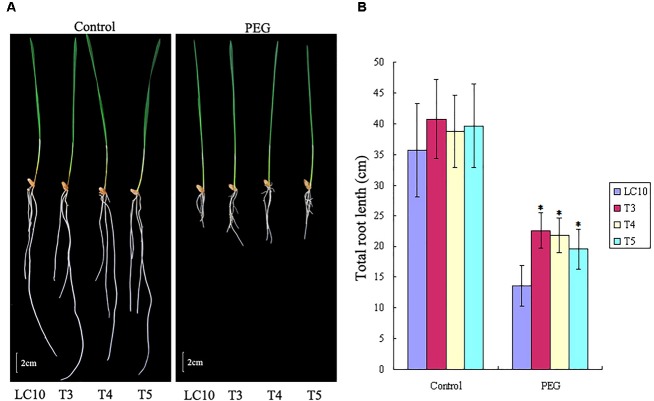
Dehydration stress tolerance assay of *TaPEPKR2* transgenic wheat seedlings. **(A)** Phenotypes of 10-day-old LC10 and transgenic lines overexpressing *TaPEPKR2* under control and 20% PEG conditions. **(B)** Total root length of 10-day-old LC10 and transgenic seedlings under stress conditions. The symbol “^∗^” indicates significance at *P* < 0.05.

Because *TaPEPKR2* was induced by 20% PEG treatment, this suggested that *TaPEPKR2* may be involved in dehydration stress tolerance. As expected, *TaPEPKR2* transgenic lines showed significantly higher total root lengths in the presence of 10% PEG than WT (Figure 4). These results indicate that the overexpression of *TaPEPKR2* in wheat conferred dehydration stress tolerance.

### *TaPEPKR2* Overexpression in Dicotyledonous *Arabidopsis* Also Enhanced Tolerance to Heat and Dehydration Stresses

**FIGURE 5:**
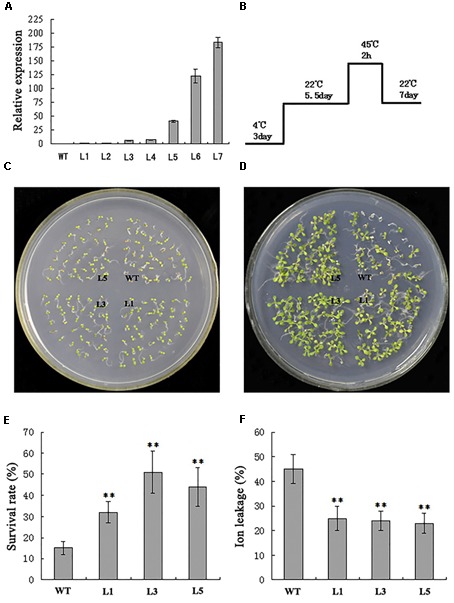
Effect of *TaPEPKR2* overexpression on heat tolerance in transgenic *Arabidopsis*. **(A)** Real-time PCR of transgenic *Arabidopsis* lines overexpressing *TaPEPKR2*. L1–L7 are seven individual *TaPEPKR2* transgenic lines. Relative *TaPEPKR2* expression in transgenic line L1 was set as the control. Data are the means of three replicates ± SD. **(B)** The heat treatment regime. **(C)** Seedlings grown for 5.5 days at 22°C were used as the control. **(D)** Phenotypes of WT, L1, L3, and L5 after heat treatment and recovery at 22°C for 7 days. **(E)** Survival rates of three *TaPEPKR2* transgenic lines (L1, L3, and L5) and WT after heat treatment. Data are the mean of three replicates ± SD (*n* = 60 for each experiment). **(F)** Ion leakage assay of the seedlings in panel **(D)** at 42°C for 1 h. Data are the means ± SD of three replicates; ^∗∗^*P* < 0.01 (Student’s *t*-test).

To characterize the biological functions of *TaPEPKR2*, we overexpressed it in *Arabidopsis*. RT-qPCR results indicated that all seven transgenic lines showed high expression levels of *TaPEPKR2*, with highest expression detected in transgenic line L7 (Figure 5A). To further validate the function of *TaPEPKR2* in plant tolerance to heat and dehydration stresses, we observed the phenotypes of *TaPEPKR2* transgenic *Arabidopsis* at various developmental stages but found no morphological differences between transgenic lines and WT (data not shown).

Seven-day-old *TaPEPKR2-OE* and WT seedlings grown at 22°C were subjected to heat treatment at 45°C for 2 h (Figure 5B). Before the treatment, there was no evident difference between transgenic and WT lines (Figure 5C), whereas after the treatment, the survival rate of transgenic lines L3 and L5 was much higher than that of WT seedlings (Figures 5D,E). Electrolyte leakage of detached leaves was evaluated under heat stress conditions. The results showed that detached leaves of WT plants had released more electrolytes than transgenic leaves (Figure 5F). These results indicate that *TaPEPKR2* overexpression in *Arabidopsis* also enhances thermotolerance.

**FIGURE 6:**
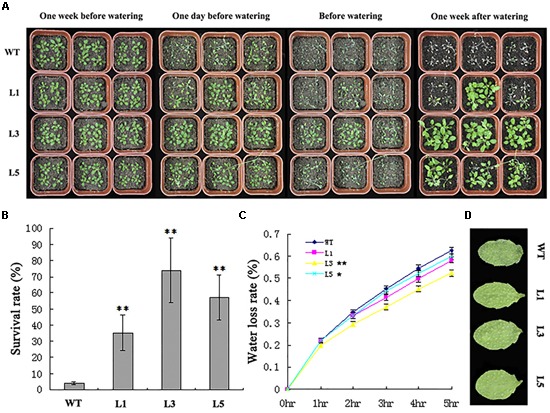
*TaPEPKR2* transgenic plants show improved dehydration tolerance. **(A)** WT and *TaPEPKR2* transgenic seedlings grown in soil were subjected to water deprivation for 25 days, then re-watered for ∼1 week. **(B)** Survival rates of WT and *TaPEPKR2* transgenic plants after dehydration treatment. Data are the mean ± SD of three replicates (*n* = 30 for each experiment); ^∗∗^*P* < 0.01 (Student’s *t*-test). **(C)** Relative water loss rate of WT and *TaPEPKR2* transgenic plants. Data are the mean ± SD of three replicates; ^∗^*P* < 0.05, ^∗∗^*P* < 0.01 (Student’s *t*-test). **(D)** Leaf wilting of detached leaves from WT and transgenic plants after 5 h.

To further investigate the performance of transgenic *TaPEPKR2Arabidopsis* plants, lines L1, L3, and L5 were selected for dehydration tolerance tests before bolting (Figure 6). After dehydration stress (1 week after watering), around 95% of WT seedlings died, whereas 35–70% of *TaPEPKR2* transgenic seedlings survived (Figures 6A,B). We further validated relative water loss rates of detached leaves (Figures 6C,D). Compared with WT plants, lines L1, L3, and L5 displayed lower relative water loss rates, and the final relative water content of detached rosette leaves from lines L3 and L5 was significantly higher than that of controls. These results indicated that the overexpression of *TaPEPKR2* resulted in dehydration tolerance in *Arabidopsis*.

## Discussion

In the present study, we identified a wheat PEP carboxylase kinase-related kinase gene, *TaPEPKR2*, that we showed to be involved in both heat and dehydration stress tolerance. SMART analysis revealed that the TaPEPKR2 protein sequence possessed a S_TKc motif (Supplementary Figure S1). These results suggested that *TaPEPKR2* is a typical serine–threonine protein kinase gene. Sequence alignment indicated that *TaPEPKR2* shares 81.98% similarity with *OsPEPKR2*, 81.46% similarity with *ZmPEPKR2*, and 53.18% similarity with *AtPEPKR2*, suggesting that it is highly conserved. The S_TKc motif of PEPKRs showed the highest homology with PPCKs, however, PEPKR proteins contain both N- and C-terminal extensions without the PPCK superfamily (Hrabak et al., 2003). These results indicated that PEPKR and PPCK functions have commonalities but also exhibit differences.

The upstream sequence of *TaPEPKR2* was analyzed for the presence of *cis*-acting elements by using the PlantCARE database (Supplementary Table S2). We found the promoter region of *TaPEPKR2* contained the putative CGTCA-motif/TGACG-motif, ABRE, Skn-1-motif/GCN4, TC-rich repeats, GARE-motif/TATC-box, TCA-element, MBS and WUN-motif, indicating the expression of *TaPEPKR2* could be regulated by several types of stresses, including abiotic and biotic stresses. The expression pattern is a direct indication of gene involvement in developmental events. In this study, *TaPEPKR2* expression was induced during heat and dehydration stress (Figure 2); however, there was no report of *TaPEPKR2* homologs from other species previously. The expression pattern of *TaPEPKR2* homologous genes was analyzed in the *Arabidopsis* Weigelworld Database^2^ and Plant Expression Database^3^. In *Arabidopsis, AtPEPKR2* was induced by heat and drought stress within a small range. In durum wheat cv. Cappelli, neither heat stress nor drought stress alone could induce *TdPEPKR2* expression, however, this was achieved by a combination of the two. In durum wheat cv. Ofanto, *TdPEPKR2* was induced by heat stress within a small range, but not by drought stress. However, their combination significantly induced *TdPEPKR2* expression. In common, wheat drought sensitive cv. A24-39, *TaPEPKR2* was weakly induced by drought stress, while in common wheat drought tolerant cv. Y12-3, *TaPEPKR2* was significantly induced by drought stress (Krugman et al., 2010). In barley caryopsis, the orthologous gene of *TaPEPKR2* (probe Barley1_05454) was induced by 0.5, 3, and 6 h heat stress treatment (Mangelsen et al., 2010). The expression pattern of *TaPEPKR2* and its orthologous genes induced by high temperature and dehydration hinted the function of *PEPKR2* in heat and drought tolerance.

Our results revealed that overexpression of *TaPEPKR2* in wheat imparted tolerance to heat and dehydration stresses compared to wild type plants. It was fist report about function of *PEPKRs* in plant. *PEPKRs* belonged to the CDPK-SnRK superfamily (Hrabak et al., 2003). Not surprisingly, many of these kinases have been implicated in response to both heat and/or dehydration stresses. PEPKRs also called PPCK related kinases and shared catalytic domain with PPCK. Previous studies have concluded that PPCK activity in rice positively regulates PEPC activity during exposure to osmotic stress (Feria et al., 2016; Liu et al., 2017). Furthermore, PPCK gene expression and activity are regulated by signaling molecules such as Ca^2+^ and H_2_O_2_ (Liu et al., 2017). Are there PEPRKs regulatory proteins which play important role in calcium and ROS homeostasis under abiotic stress? What potential specific substrates they have? Further studies are needed to determine the molecular mechanisms involved in enhancing heat and dehydration stresses tolerance in TaPEPKR2-OE plants. Relative expression levels of the genes functioning in ABA biosynthesis (*ABA1*, AT5G67030), signaling (*ABI3*, AT3G24650), *heat shock protein 70* (*HSP70*, AT3G12580), and *17.6A* (*HSP17.6A*, AT5G12030) were investigated in *TaPEPKR2* transgenic *Arabidopsis* plants (Supplementary Figure S3). The expressions of *ABA1* and *HSP70* were found to be unchanged, however, *ABI3* and *HSP17.6A* were elevated constitutively. These results present some clues of *TaPEPKR2* regulating ABA signaling and heat shock proteins in heat and dehydration stresses.

## Author Contributions

QS, HP, and XZ designed the research. XZ, XG, KH, FW, and XT performed the research. XZ, XG, HP, ZN, YY, ZH, and MX analyzed the data. XZ and HP wrote the paper. All authors read and approved the final manuscript.

## Conflict of Interest Statement

The authors declare that the research was conducted in the absence of any commercial or financial relationships that could be construed as a potential conflict of interest.

## Footnotes

1 http://smart.embl-heidelberg.de/

2 http://jsp.weigelworld.org/expviz/expviz.jsp

3 http://www.plexdb.org/

## Supplementary Material

FIGURE S1 The functional domain of the TaPEPKR2 protein sequence identified by the SMART program.

FIGURE S2 PCR analysis of *TaPEPKR2* transgenic wheat plants. (A) Confirmation of *TaPEPKR2* insertion in LC10 by PCR analysis of H_2_O, LC10, PC, L3, L4, and L5 transgenic plants. PC: *Ubi::TaPEPKR2* vector was used as the positive control. (B) Relative expression of *TaPEPKR2* as shown by RT-qPCR. *β-actin* was used as the internal control. Data are the mean ± SD of three independent biological replicates.

FIGURE S3 Relative expression level of *ABA1* (A), *ABI3* (B), *HSP70* (C), and *HSP17.6A* (D) in 10-day-old *TaPEPKR2* transgenic *Arabidopsis* plants, as determined by RT-qPCR. Data represent the mean of three replicates ± SD.

TABLE S1 Primer sequences used in this study.

TABLE S2 *Cis*-elements present in the promoters of *TaPEPKR2*.

FILE S1 Coding sequence of *TaPEPKR2*.
