# Supplementary figures and images for: Overexpression of the Wheat (Triticum aestivum L.) TaPEPKR2 Gene Enhances Heat and Dehydration Tolerance in Both Wheat and Arabidopsis
Source: Front Plant Sci. 2018 Nov 23;9:1710. doi: 10.3389/fpls.2018.01710 (PMC6265509; doi:10.3389/fpls.2018.01710)

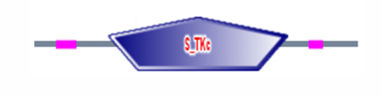

Supplement: FIGURE S1 — The functional domain of the TaPEPKR2 protein sequence identified by the SMART program. [file Image_1.jpeg]

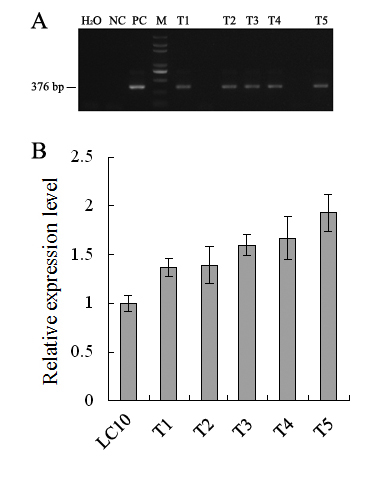

Supplement: FIGURE S2 — PCR analysis of TaPEPKR2 transgenic wheat plants. (A) Confirmation of TaPEPKR2 insertion in LC10 by PCR analysis of H2O, LC10, PC, L3, L4, and L5 transgenic plants. PC: Ubi::TaPEPKR2 vector was used as the positive control. (B) Relative expression of TaPEPKR2 as shown by RT-qPCR. β-actin was used as the internal control. Data are the mean ± SD of three independent biological replicates. [file Image_2.jpeg]

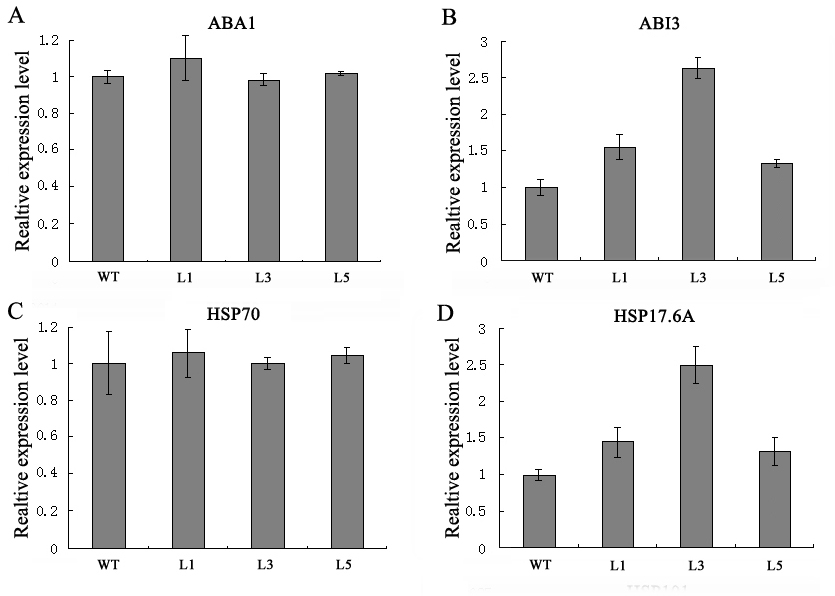

Supplement: FIGURE S3 — Relative expression level of ABA1 (A), ABI3 (B), HSP70 (C), and HSP17.6A (D) in 10-day-old TaPEPKR2 transgenic Arabidopsis plants, as determined by RT-qPCR. Data represent the mean of three replicates ± SD. [file Image_3.jpeg]
