# Supplementary material for: Overexpression of the Wheat (Triticum aestivum L.) TaPEPKR2 Gene Enhances Heat and Dehydration Tolerance in Both Wheat and Arabidopsis
Source: Front Plant Sci. 2018 Nov 23;9:1710. doi: 10.3389/fpls.2018.01710 (PMC6265509; doi:10.3389/fpls.2018.01710)
Supplement: TABLE S1 — Primer sequences used in this study. [file Table_1.docx]

**Table S1.** Primers used in this paper.

| **Number** | **Name** | **Sequence (5′-3′)** |
| --- | --- | --- |
| 1 | *TaPEPKR2*-L | ATGGAGTCGCTGCCGCGGAAG |
| 2 | *TaPEPKR2*-R | ATTGGGGTCATGTCAGAACGCC |
| 3 | Q-*TAPEPKR2*-L | TTTGCTCACCAAGACTGGC |
| 4 | Q-*TAPEPKR2*-R | CCGTCCAAGAACTGACATTGA |
| 5 | *TaPEPKR2*- L2 | TTTGGATTGGCAGCACGAGT |
| 6 | *TaPEPKR2*- R2 | GGAGTTACAGCCTTCAGGGT |
| 7 | *β-actin*-L | GGAATCCATGAGACCACCTAC |
| 8 | *β-actin*-R | GACCCAGACAACTCGCAAC |
| 9 | Q-*ABA1*-F | *GCTATGAAGGTGATCTGCTTGTGG* |
| 10 | Q-*ABA1*-R | *TTCATACCATTTGGAGCATCAGC* |
| 11 | Q-*ABI3*-F | *CACAGCCAGAGTTCCTTCCTTTACT* |
| 12 | Q-*ABI3*-R | *TAGTTGCTGAGGAACACAAACGG* |
| 13 | Q-*HSP70*-F | *AGGAGCTCGAGTCTCTTTGC* |
| 14 | Q-*HSP70*-R | *AGGTGTGTCGTCATCCATTC* |
| 15 | Q-*HSP17.6A*-F | *GGTGAGTGGCAAAAGACAGA* |
| 16 | Q-*HSP17.6A*-R | *AAACTTCCCCATCCTCCTCT* |
| 17 | 18S rRNA-L | ACATCCAAGGAAGGCAGCA |
| 18 | 18S rRNA-R | TAAGACCAGGAGCGTATCGC |
