# Supplementary material for: Overexpression of the Wheat (Triticum aestivum L.) TaPEPKR2 Gene Enhances Heat and Dehydration Tolerance in Both Wheat and Arabidopsis
Source: Front Plant Sci. 2018 Nov 23;9:1710. doi: 10.3389/fpls.2018.01710 (PMC6265509; doi:10.3389/fpls.2018.01710)
Supplement: TABLE S2 — Cis-elements present in the promoters of TaPEPKR2. [file Table_2.docx]

**Table S2.** A selection of putative cis-acting elements identified in the PlantCARE database is indicated.

| Cis-acting element | Function | Number |
| --- | --- | --- |
| CGTCA-motif /TGACG-motif | cis-acting regulatory element involved in the MeJA-responsiveness | 8 |
| ABRE | cis-acting element involved in the abscisic acid responsiveness | 4 |
| Skn-1-motif/ GCN4 | cis-acting regulatory element required for endosperm expression | 4 |
| TC-rich repeats | cis-acting element involved in defense and stress responsiveness | 2 |
| GARE-motif/ TATC-box | gibberellin-responsive element | 2 |
| TCA-element | cis-acting element involved in salicylic acid responsiveness | 1 |
| MBS | MYB Binding Site | 1 |
| WUN-motif | wound-responsive element | 1 |
