# Supplementary material for: Overexpression of the Wheat (Triticum aestivum L.) TaPEPKR2 Gene Enhances Heat and Dehydration Tolerance in Both Wheat and Arabidopsis
Source: Front Plant Sci. 2018 Nov 23;9:1710. doi: 10.3389/fpls.2018.01710 (PMC6265509; doi:10.3389/fpls.2018.01710)
Supplement: FILE S1 — Coding sequence of TaPEPKR2. [file Data_Sheet_1.docx]

**File S1:** Coding sequence of *TaPEPKR2*.

1 ATGGAGTCGC TGCCGCGGAA GCGCAAGGGC GCGCGCTCCC TCGCCGGCTC CCTCCACGAC

61 GCCTCCGCGG ACCGCAAGCG GACCTGCCGG GAGCGGAAGC CGCGCCCCGA CAAGAAGAAA

121 AAGAAGCCCT CCGCCGCCGG CGATGACGCC GCCACCGCCT CCGGCCGGGG CGGCGTGGTC

181 ATGACGGCGC CACCGGCCAG CGGCCGGGCC ACCCCGGACA GCCCCGGCCG GGGCCTCAAG

241 CGCAAGGTCG GCTGCATCGA GTCCGCCACG CGCATAGGCC GCAAGAAGCG CCTCGAGACC

301 GAGTACGAGC TCGGCGACGA GATCGGCCAG GGCAAGTTCG GCTCCGTCCG GATCTGCCGC

361 GCCAAGGCCG GCGGCGAGGA GTTCGCCTGC AAGGCGCTCC CCAAGAACGG CGAGGAGACG

421 GTCCACCGCG AGGTCGAGAT CATGCAGCAC CTCTCCGGCC ACCCCGGCGT CGTCACGCTC

481 AAGGCCGTCT TCGAGGACGC CGACAAGTTC TACCTCGTCA TGGAGCTCTG CAGCGGCGGC

541 CGCTTGCTCG ACGAGATGGC CAGGGACGGC ACCTTCTCCG AGCGGCGAGC CGCCCTCGTC

601 ACCAAGGATC TAATGTCGGT CGTCAAGTAC TGCCACGAAA TGGGCGTCAT CCACAGGGAC

661 ATTAAGCCGG AGAATATTTT GCTCACCAAG ACTGGCAAGA TGAAACTAGC TGATTTTGGA

721 TTGGCAGCAC GAGTTACTAA CGGTCAGAAA TTGTCTGGCG TTGCTGGGAG CCCAGCCTAT

781 GTGGCGCCTG AGGTGTTGTC AGGAAGCTAT TCTGAGAAAG TAGACATATG GGGTGCTGGG

841 GTGCTCCTCC ATGTACTACT GCTTGGTTCA CTTCCATTTC AAGGGGGCTC TCTGGAAGCT

901 GTCTTTGAAG CTATAAAGAC AGTTGAGCTT GATTTCAACA GCGGTCCATG GGAATCAATG

961 TCAGTTCTTG GACGGGATCT TATAAGTCGA ATGTTGGATC GAGACGTCTC TTCTAGAATG

1021 ACTGCTGATC AAGTTCTTTG TCATCCATGG GTGTTGTTTT ACACGGAATG TACCCTGAAG

1081 GCTGTAACTC CTAATGTCAC TAACCAGATT GTAGCACCCA AAATTCCATG GGACAGAATT

1141 AGATCACATT CTGAGTCGTC AGCTTCAGAT TCGTCGAGCC AGAGGTCGGA GGACCAGGAT

1201 GAATGTGGCA TAGTCGACGC ACTGACTGCG GCAATAACAC ATGTTAGAAT ATCGGAGCCG

1261 AAAAGAACCC GGCTTTGCAG CCCTGGCATT CCCATACAGC AGGAATGCTC CTCAAACTTA

1321 AAGAGCAACC TGTGCACGGC GTTCTGA
